# Role of Reactive Oxygen Species and Hormones in Plant Responses to Temperature Changes

**DOI:** 10.3390/ijms22168843

**Published:** 2021-08-17

**Authors:** Amith R. Devireddy, Timothy J. Tschaplinski, Gerald A. Tuskan, Wellington Muchero, Jin-Gui Chen

**Affiliations:** 1 Biosciences Division, Oak Ridge National Laboratory, Oak Ridge, TN 37831, USA; devireddyar@ornl.gov (A.R.D.); tschaplinstj@ornl.gov (T.J.T.); tuskanga@ornl.gov (G.A.T.); 2 Center for Bioenergy Innovation, Oak Ridge National Laboratory, Oak Ridge, TN 37831, USA

**Keywords:** heat stress, cold stress, ROS, hormone, signal transduction, signal integration, molecular mechanisms, acclimation

## Abstract

Temperature stress is one of the major abiotic stresses that adversely affect agricultural productivity worldwide. Temperatures beyond a plant’s physiological optimum can trigger significant physiological and biochemical perturbations, reducing plant growth and tolerance to stress. Improving a plant’s tolerance to these temperature fluctuations requires a deep understanding of its responses to environmental change. To adapt to temperature fluctuations, plants tailor their acclimatory signal transduction events, and specifically, cellular redox state, that are governed by plant hormones, reactive oxygen species (ROS) regulatory systems, and other molecular components. The role of ROS in plants as important signaling molecules during stress acclimation has recently been established. Here, hormone-triggered ROS produced by NADPH oxidases, feedback regulation, and integrated signaling events during temperature stress activate stress-response pathways and induce acclimation or defense mechanisms. At the other extreme, excess ROS accumulation, following temperature-induced oxidative stress, can have negative consequences on plant growth and stress acclimation. The excessive ROS is regulated by the ROS scavenging system, which subsequently promotes plant tolerance. All these signaling events, including crosstalk between hormones and ROS, modify the plant’s transcriptomic, metabolomic, and biochemical states and promote plant acclimation, tolerance, and survival. Here, we provide a comprehensive review of the ROS, hormones, and their joint role in shaping a plant’s responses to high and low temperatures, and we conclude by outlining hormone/ROS-regulated plant responsive strategies for developing stress-tolerant crops to combat temperature changes.

## 1. Introduction

In natural habitats, plants are often exposed to multiple stresses individually or in combination. These stressors, either biotic or abiotic, are potentially unfavorable, interfering with and attenuating the plant’s normal biological functions [1]. Temperature is one of the primary physical parameters that govern a plant’s health. Atmospheric temperatures have increased over the last two decades and are expected to continue to rise 1.0 to 1.7 °C by 2050 and 4.0 to 5.0 °C by the end of the 21st century, at which point, high temperature stress will become one of the most frequent abiotic stresses confronted by plants [2,3,4]. In contrast, low temperatures limit the geographical distribution of plant species and negatively affect their biological functions. In total, temperature extremes, together with other abiotic stresses, reduce average crop yields by more than 50% and continue to pose a risk to agricultural and forest production [5,6,7].

By definition, any fluctuation in temperature that induces irreversible damage to the plant’s metabolism or growth is considered stress and is categorized into high temperature or low-temperature stress [8]. High temperature stress results in many cellular and physiological changes, e.g., protein denaturation, loss of membrane integrity, reduced cellular function, and reduced plant growth. High temperatures also result in drought stress, another global issue. Drought and high temperature stress conditions individually or in their combination cause oxidative and osmotic stress, contributing to reduced plant growth and development [9,10].

The temperature is initially sensed by plant thermosensors, including calcium channels in the plasma membrane, histone sensors in the nucleus, reactive oxygen species (ROS) in the cell, and denatured proteins in the endoplasmic reticulum and cytosol. These sensors trigger signaling pathways that ultimately result in the expression of heat stress-responsive genes, contributing to plant thermotolerance [11,12]. In addition, plants have developed a wide range of survival strategies to overcome temperature changes. The ability of the plants to survive abrupt temperature increases is known as basal thermotolerance [11]. Plants also possess acclimatory responses known as acquired thermotolerance, where the pre-exposure to sublethal heat stimuli can aid in cellular reprogramming and increase the thermotolerance [13,14].

Low temperature stresses negatively impact plant growth and development by inhibiting the activity of the metabolic enzymes, altering the gene expression, and influencing the plant metabolism and transcriptome, thus delaying many developmental processes and growth [15]. Cold stress includes chilling stress (0–20 °C) and freezing stress (<0 °C), and stress tolerance varies depending on the plant species and temperature ranges [16]. Interestingly, high and low temperatures share some common responses, including changes in the membrane fluidity, alterations in the levels of plant hormones, the production and accumulation of ROS, calcium, nitric oxide (NO) signaling, Mitogen-activated protein kinase (MAPK) signaling, protein sumoylation and proteasomal degradation, the reprogramming of transcriptomic or metabolomic signatures, and other stress-responsive signaling cascades [17,18]. In contrast, some of the distinct signaling components in high- and low-temperature response pathways include the up- and downregulation of transcription factors (TFs) such as heat shock factor (HSF) family TFs and Inducer of CBF expression (ICE1) TFs that regulate the heat and cold stress responses, respectively, and the formation and accumulation of secondary metabolites, sugars, and amino acids.

**Figure 1:**
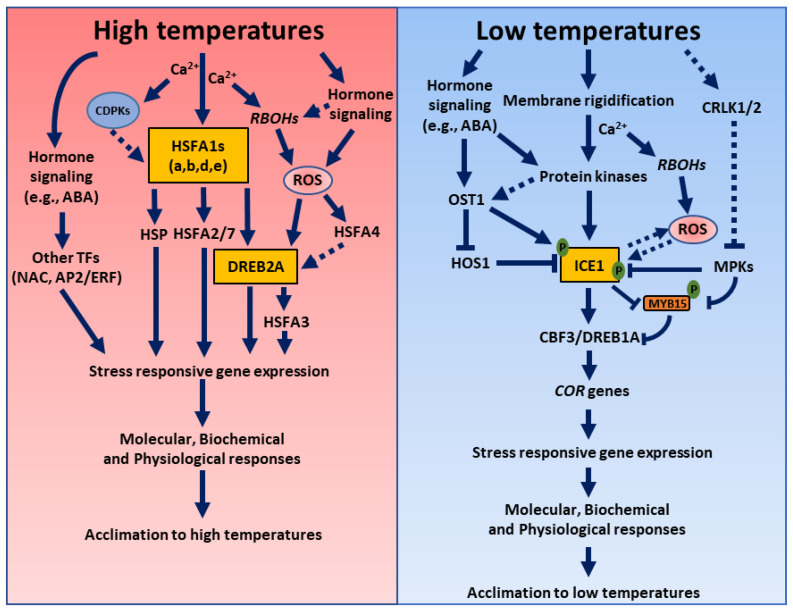
A schematic model showing coordination between various signaling components to induce acclimation responses to temperatures stress. The interplay between the ROS; hormones; and other signaling components such as Ca^2+^, CDPKs, MAPKs, and TFs determines the plant’s molecular, biochemical, metabolic and physiological responses to high or low temperatures. Left panel: Heat stress affects the membrane fluidity and simultaneously triggers hormone signaling pathways, resulting in increased cytosolic calcium, reactive oxygen species, and the activation of other cytoplasmic proteins. The intercellular calcium activates CDPKs or RBOHs, which result in ROS production. Hormones and ROS, along with other signaling components, interdependently activate multiple signaling pathways to alter the activity of the heat shock factors (e.g., HSFA1), which, in turn, activate other transcription factors like Dehydration-Responsive Element-Binding Protein2a (*DREB2A*), inducing the appropriate stress-responsive gene expression. Right panel: Low temperatures trigger plasma membrane rigidification, and activate the Ca^2+^ channels, leading to increased Ca^2+^ concentrations in the cytosol. The cytosolic calcium activates protein kinases such as CDPKs that, in turn, phosphorylate the transcription factor ICEAlternatively, ICE1 can be phosphorylated by ABA-induced OSTICE1 activates the expression of the *CBF* genes by directly binding to their promoters at low temperatures, inducing the appropriate *COR* gene expression. Some of the common features between high- and low-temperature response pathways include changes in the membrane fluidity, alterations in the levels of plant hormones, the production and accumulation of ROS, calcium, NO and MAPK signaling, protein sumoylation and proteasomal degradation, the reprogramming of transcriptomic or metabolomic signatures, and other stress-responsive signaling cascades. Distinct signaling components in high- and low-temperature response pathways include TFs, proteins, stress-responsive genes, the formation and accumulation of secondary metabolites, sugars, amino acids, etc. ABA, abscisic acid; AP2/ERF, Apetala2/Ethylene Responsive Factor; Ca^2+^, calcium; CBF, C-repeat-binding factor; CDPK, calcium-dependent protein kinase; *COR*, cold-response genes; CRLK, calcium/calmodulin-regulated receptor-like kinases; DREBs, dehydration response element-binding factors; *HOS1*, HIGH EXPRESSION OF OSMOTICALLY RESPONSIVE GENE1; HSP, heat shock proteins; HSF, HEAT SHOCK FACTOR; ICE1, inducer of CBF expression 1; MPKs, mitogen-activated protein kinase; MYB, Myb-like transcription factors; NAC, NAM, ATAF, and CUC family TF; OST1, OPEN STOMATA 1; RBOHs, respiratory burst oxidase homologs; ROS, reactive oxygen species; and SnRK2, SNF1-related protein kinase 2.

Plant responses to abnormal or extreme temperature changes are primarily mediated by plant hormones that control and mediate complex stress-adaptive signaling cascades and induce heat or cold stress responses [19,20]. One of the mechanisms by which plant hormones induce thermotolerance is by inducing ROS production, activating NADPH oxidases, and/or by altering the redox signaling that regulates various cellular and physiological responses in response to temperature changes [21,22]. The extensive crosstalk between the ROS and hormone signaling pathways aids in plant development and acclimation responses (Figure 1) [22,23,24,25,26]. A few studies also suggest that ROS can mediate signaling crosstalk events between different hormones, resulting in improved thermotolerance [27,28,29,30]. In addition, plant hormones and ROS interact with other key signaling molecules or components, such as TFs, Ca^2+^, NO, kinases such as calcium-dependent protein kinase (CDPKs) and mitogen-activated protein kinases (MAPKs), and phosphatases, to orchestrate the required molecular and physiological responses to survive extreme temperatures and regulate plant developments [11,31,32,33]. Although several studies have demonstrated the role of ROS as secondary messengers in hormone signal transduction pathways, the in-depth and precise mechanisms of ROS and hormone interactions during temperature stress remain to be fully elucidated. In this review article, we highlight the recent and early studies on the plant tolerance to temperature changes, especially mediated through the underlying signal transduction pathways of the ROS, hormones, and their integration. From this perspective, we provide insights on strategies to improve crop tolerance to temperature stress that will aid in coping with the global temperature fluctuations.

## 2. ROS Signaling

In contrast to its damaging effects and role in thermotolerance, ROS in their optimal levels serve as signaling molecules generated in response to stress perception by stress sensors [11,22,34]. As signaling molecules, ROS are distributed in all the metabolically active plant tissues and are controlled by the ROS gene network [11,35,36]. During temperature stress—specifically heat stress, although the chloroplast is the major site of ROS production in plants—the signaling ROS are known to be mediated by calcium or by the activation of NADPH oxidases at the plasma membrane and act as a heat stress signal transducer [36,37,38,39,40].

ROS, along with other signals such as Ca^2+^, are also involved in the long-distance systemic signaling required for the activation of systemic acquired acclimation in response to heat or other abiotic stresses [21,41,42,43,44,45]. For example, upon the perception of heat stress by sensors, plant hormones such as abscisic acid (ABA) and jasmonic acid (JA) trigger ROS production, which, in turn, activates the RBOH proteins in the neighboring cells to further generate ROS, initiating a systemic signal (the ROS wave) [38,46]. The hormone-triggered ROS move via cell-to-cell propagation, forming an amplification loop, and activate the systemic acquired acclimation responses [33,43,45]. Studies have shown that the local application of heat or cold stimuli induced similar stress transcriptional responses in both local and systemic tissues that were shown to be ROS wave-dependent [42,45,47]. Similarly, ROS activate calcium channels, which, in turn, activate the Two Pore Channel1 (*TPC1*), a vacuolar calcium channel that transports vacuolar-stored Ca^2+^, resulting in the activation of the respiratory burst oxidase homolog D (RBOHD) proteins [48]. This mechanism occurs in a feedback loop activating ROS and calcium, thus inducing a whole-plant acclimation response to high or low temperatures [36,41,42,45].

## 3. High Temperatures Stress

Heat stress affects the membrane fluidity, resulting in increased cytosolic calcium, which initiates downstream calcium signaling mediated by the plasma membrane-localized Cyclic Nucleotide Gated Channel (CNGC) family proteins or several Ca^2+^ sensors like calmodulins (CaMs), including CMLs (CaM-like proteins), calcineurin B-like proteins (CBLs), CDPKs, and Ca^2+^ binding proteins [49]. The Ca^2+^/CaM-binding protein kinase AtCBK3 participates in heat stress responses by targeting AtHSFA1a [50]. On the other hand, the HS-initiated Ca^2+^ signaling is transduced via RBOH proteins, initiating the ROS burst at the apoplast. The generated ROS by RBOHs is transported into the cell via the aquaporins to trigger the further release of Ca^2+^ by TPC1 channels to regulate different signaling and stress responses [21,51]. Thus, calcium-ROS signaling, along with other hormone signaling components, activates multiple downstream signaling pathways that regulate the heat shock transcription factors (HSFs) via post-translational modifications, thereby inducing heat stress responses (Figure 1).

### 3.1. Role of ROS during High Temperatures Stress

The perception of temperature changes by the plant receptors or sensors initiates several complex signaling networks that result in decreased membrane thermostability; higher malondialdehyde (MDA) accumulation; and the generation of ROS such as hydrogen peroxide (H_2_O_2_), singlet oxygen (^1^O_2_), superoxide (O_2_^−^), or hydroxyl radical [11,52]. In plants, ROS are mainly produced by the Respiratory Burst Oxidase Homolog (RBOH) proteins, the plasma membrane-localized NADPH oxidases, and several peroxidases that are controlled by the ROS gene network [11,35,36]. The ROS generated in plants are decoded by various ROS sensors, including Glutathione peroxidases (GPXs), plasma membrane-localized receptor-like kinases (RLKs), Cys-rich receptor-like kinases (CRKs), serine/threonine protein kinase (OXI1), cyclic nucleotide-gated channels activated by heat stress, and redox response transcription factors like HsfA4a and, thereafter, initiate stress-specific signals that induce the required gene expression and protein synthesis [11,53,54,55,56,57]. However, if uncontrolled, ROS overproduction results in oxidative stress that can damage proteins, biomolecules, and membranes, thereby affecting the photosynthetic machinery and overall leaf physiology [58,59,60]. For example, exposing chickpea or rice plants to heat stress of (32/20 °C Day/Night, 7 d) resulted in a 6.5-fold increase of H_2_O_2_ accumulation and resulted in oxidative damage compared to the controls [61,62]. Nevertheless, many studies have revealed the importance of ROS and its regulatory systems at various stages of plant development in response to heat stress [63,64]. ROS were shown to regulate the plant’s basal and acquired thermotolerance. In Arabidopsis, Davletova et al. and Miller et al. showed that mutants deficient in the antioxidant pathways, such as ascorbate peroxidase 1 (*apx1*), were defective in basal thermotolerance and showed a greater sensitivity to heat stress [65,66]. Davletova et al. (2005) also showed, using knockout (KO) mutants of cytosolic ascorbate peroxidase, an ROS scavenging enzyme and that heat shock transcription factor 21 (HSF21/AtHSFA4) is involved in H_2_O_2_ stress sensing, suggesting a close link between the HSF and ROS [65]. A few other major ROS responsive TFs include MYB domain protein 44 (MYB44), Heat Stress transcription factor A-4A (HSFA4A), and Ethylene Responsive Element-Binding Factor 6 (*ERF6*) [53,67,68,69,70,71]. Similarly, heat stress induction has been shown to trigger the expression of heat shock proteins HSP17.6 and HSP18.2 in Arabidopsis cell cultures. On the other hand, the application of ROS inhibitors such as diphenyleneiodonium chloride significantly reduced the *HSP* gene expression, suggesting that heat stress induced H_2_O_2_ is required for the effective expression of heat shock genes in Arabidopsis [70,72].

In response to the excess production of ROS under high temperatures, plants not only initiate heat stress (HS) responsive pathways but also induce expression of stress-related and antioxidant proteins that result in higher antioxidant activities of ascorbate peroxidase (APX), catalase (CAT), or superoxide dismutase (SOD), thereby reducing the negative effects of oxidative damage caused by ROS [35]. Studies using the KO mutants of ROS-scavenging enzymes were shown to be HS-sensitive, suggesting that they are necessary for the detoxification of excess ROS under high temperatures. Similarly, in tomatoes, the HS-induced negative effects of ROS, such as reduced growth, were reversed when treated with a ROS-scavenging antioxidant ascorbic acid. Studies using *phyB* mutants have suggested that the light priming of ROS detoxification via *APX2* is a key component of thermotolerant adaptation [73,74,75]. Another study reported that an RNA-binding protein, flowering control locus A, is required to induce thermotolerance by triggering antioxidant accumulation under heat stress conditions [76]. Recent studies have also shown that miRNAs, such as miR398, regulate the heat response TF by negatively regulating several ROS-scavenging enzymes, resulting in an ROS accumulation that triggers HSFA1 to induce heat stress responses [77,78,79]. For instance, in rice, the NAC transcription factor gene *SNAC3* (ONAC003, LOC_Os01g09550) modulates the H_2_O_2_ homeostasis by controlling the expression of ROS-associated genes, thus serving as a positive regulator under high temperatures [80]. In total, the evidence suggests that ROS-scavenging systems prevent an excess accumulation of toxic ROS and regulate the thermotolerance in plants [81,82]. Likewise, the studies using ROS biosynthesis mutants, such as NADPH oxidase (*atrbohB* and *atrbohD*), showed a reduced thermotolerance due to the lower levels of ROS, indicating that plants are required to maintain ROS at the optimal levels for attaining thermotolerance [63,83]. Similarly, studies have shown that priming plants with heat improved the antioxidant capacity, accumulated lower ROS, and delayed the ROS-induced cell death [84,85]. Heat priming also improved photosynthesis by enhancing stomatal conductance and by synthesizing secondary compounds with antioxidative characteristics that helped maintain the leaf membrane integrity under high temperature stress [86,87]. Taken together, these studies suggest that ROS regulate the trade-off between the growth, acclimation, or defense responses at altered temperatures [88,89].

### 3.2. Role of Hormones during High Temperatures Stress

The plant responses to abnormal or extreme temperature changes are primarily mediated by plant hormones, including abscisic acid (ABA), brassinosteroids (BRs), cytokinins (CKs), salicylic acid (SA), jasmonic acid (JA), and ethylene (ET). These hormones control and mediate complex stress-adaptive signaling cascades and induce stress-response TFs [19,20]. Various studies on the transcriptional regulation of heat and cold via TFs have uncovered both ABA-dependent and ABA-independent components and pathways, such as ABA-responsive element-binding protein/ABA-binding factor (AREB/ABF) or cold-binding factor/dehydration responsive element-binding transcription factors (CBF/DREB) [17,90,91,92].

#### 3.2.1. Role of ABA and JA during High Temperatures

**Figure 2:**
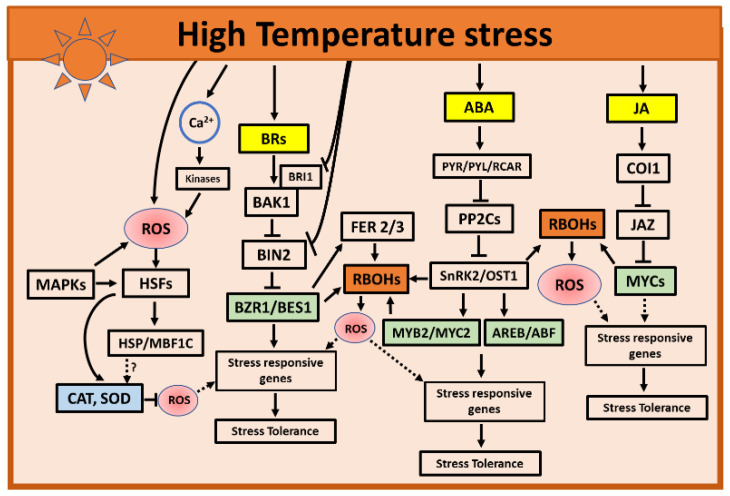
Selected hormone and ROS interactions in response to high-temperature stress. During high temperatures, ABA-regulated kinases such as SnRK2 or TFs activate RBOHs to generate ROS. Similarly, in the BR-signaling pathway TF BZR1 activates RBOHs to generate ROS. Along with TFs, the generated ROS may activate and induce the expression of stress-responsive genes to orchestrate the appropriate molecular and physiological acclimation responses to high temperatures. Other plant hormones such as ET, SA, and CKs may also play roles in inducing stress responses to high temperatures. Only ABA, BRs, and JA were selected to be presented in the mode due to the availability of a relatively large amount of data supporting their roles in ROS interactions in response to high-temperature stress. Solid arrows and blocked arrows indicate confirmed positive and negative regulations. Dashed lines indicate potential interactions. The hormones are indicated in yellow, transcription factors in green, ROS in red, and ROS-producing and -scavenging enzymes are indicated in brown and blue, respectively. ABA, abscisic acid; AREB/ABF, ABA-responsive element-binding protein/ABA-binding factor; BR, brassinosteroid; BAK1, BRI1-ASSOCIATED KINASE1; BES1, BRI1-EMS SUPPRESSOR1; BIN2, brassinosteroid insensitive 2; BRI1, BRASSINOSTEROID INSENSITIVE1; BZR1, BRASSINAZOLE-RESISTANT1; Ca^2+^, calcium; CAT, catalase; COI1, CORONATINE-INSENSITIVE 1; FER, FERONIA; HSF, HEAT SHOCK FACTOR; HSP, heat shock proteins; JA, Jasmonic acid; JAZ, JASMONATE-ZIM-DOMAIN; MAPK, mitogen-activated protein kinase; MBF1C, MULTIPROTEIN BRIDGING FACTOR 1C; MYB/C, MYB/C transcription factors; OST1, OPEN STOMATA 1; PP2C, protein phosphatase 2Cs; PYR/PYL/RCAR, pyrabactin resistance protein or PYR-like proteins; RBOHs, respiratory burst oxidase homologs; ROS, reactive oxygen species; SnRK2, SNF1-related protein kinase 2; and SOD, superoxide dismutase.

Phytohormone ABA is one of the extensively studied hormones involved in regulating heat stress responses and is required during most of the developmental stages to induce thermotolerance. Upon the perception of heat stress, ABA is released and perceived by the PYR/PYL receptors to form the ABA-PYR/PYL complex that interacts and suppresses the activity of protein phosphatase 2Cs (PP2Cs). This interaction releases (SnRK2)/Open Stomata 1 (OST1), a positive regulator of the ABA response. The released SnRK2s autophosphorylate and trigger the activity of different TFs, such as heat shock transcription factors (HSF), MYB1, or ABA-Responsive Promoter Elements (ABREs)-Binding Factors (ABFs) (AREB/ABFs), to induce heat stress responses (Figure 2) [21,93]. ABA is required during most of the developmental stages to induce thermotolerance. During the reproductive stage in Arabidopsis, ABA was shown to induce the *SPL* transcription factors conferring thermotolerance [94]. Heat stress induces a rapid increase in the endogenous ABA concentration and enhances the antioxidant ability to confer heat tolerance in plants [95,96,97]. For example, Arabidopsis and tomato mutant plants, deficient in ABA biosynthesis and signaling, exhibited heat-sensitive responses and had lower photochemical efficiency than the wild type [98]. Likewise, the plants treated with ABA showed a higher accumulation of H_2_O_2_ that mediated the induction of heat tolerance [99]. Microarray studies, using Arabidopsis, suggest that ABA can induce thermotolerance by inducing the expression of HSFA6b, a class A HSF [100]. In line with these findings, promoter mutagenesis analyses in wheat showed that transcriptional activator TaHsfC2a-B activates heat protection genes via the ABA-mediated pathway, and its overexpression improved the thermotolerance in wheat grains [97]. ABA also induces other TFs, like HSF6b and SQUAMOSA Promoter-Binding Proteinlike (SPL), required to acquire thermotolerance [94,100]. The activated HSF6b then directly binds to the promoter of *Dehydration-Responsive Element-Binding Protein2a*, enhancing its expression and, thus, inducing thermotolerance. Similarly, the TFs SPL1 and SPL12 confer thermotolerance via PYL-mediated ABA signaling [94,100].

Like ABA, plants have been shown to accumulate JA and its precursors OPDA or MeJA in response to heat stress [101,102]. Studies in JA biosynthesis or signaling mutants, such as *jar1* or *coi1*, displayed lower heat tolerances, suggesting that JA is involved in regulating plants’ basal thermotolerance [103]. In contrast, pretreating Arabidopsis plants with MeJA upregulated the antioxidant enzyme activity and conferred heat tolerance, suggesting that jasmonic acid is required for acquiring thermotolerance [103,104]. At the molecular level, JA transcriptionally induces WRKY or MYC TFs to induce thermotolerance [102,105,106,107].

#### 3.2.2. Role of BRs during High Temperatures

The perception of BR by its receptor BRASSINOSTEROID INSENSITIVE1 (BRI1) facilitates the interaction of the active BR1 with a coreceptor BRI1-ASSOCIATED KINASE1 (BAK1). This interaction positively regulates the BR-signaling components BRASSINAZOLE-RESISTANT1 (BZR1) and BRI1-EMS SUPPRESSOR1 (BES1) to enhance the expression of heat-responsive genes (Figure 2) [108]. For example, at high temperatures, the accumulation of BES1 and BZR1 increases, and BZR1 binds to the E-box and G-box elements of the *PHYTOCHROME INTERACTING FACTOR 4* (*PIF4*) promotor to regulate its expression. This interaction induces the expression of growth-promoting genes, thus regulating the thermomorphogenesis [109]. Additionally, thermomorphogenic genes are activated by the abundant levels of the BES1-PIF4 complexes, highlighting the roles of BR-induced TFs in attaining thermotolerance [110,111]. A recent study in Arabidopsis also indicated that a loss-of-function of *BRI1-EMS-SUPPRESSOR 1* (*bes1*)-mediated BR signaling, exhibited the most sensitive characteristics to heat stress, such as a lower PSII photochemistry efficiency (*Fv/Fm*), higher lipid hydroperoxide contents, higher photoinhibition, and photo-oxidative stress compared to the wild type [112]. In barley (*Hordeum vulgare*), the mutants deficient in BR biosynthesis and signaling negatively affected the accumulation of the *HSP* transcripts and heat-shock proteins, demonstrating the role of BRs in attaining plant acclimation to high temperatures [113,114]. In addition, BRs were shown to be involved in signaling crosstalks with other hormones to coordinate stress responses [23]. For example, BRs were shown to interact and enhance the endogenous level of ABA in *Chlorella vulgaris* by regulating ABA biosynthesis, thus enhancing the tolerance to heat [115,116]. Taken together, these results highlight the role of BR signaling in attaining heat stress acclimation in plants.

#### 3.2.3. Role of ET during High Temperatures

Ethylene is another hormone that is involved in the heat stress response and modulates the plant growth and development through transcription factor ETHYLENE RESPONSE FACTOR 74 (*ERF74*). Studies using overexpression lines of *ERF74* showed increased ROS-dependent dichlorofluorescein fluorescence and displayed enhanced basal thermotolerance, whereas the KO mutants accumulated lower ROS levels and showed reduced basal thermotolerance, suggesting that *ERF74* possibly regulates the RBOHD expression at high temperatures [117]. In addition, it has been shown that *erf74* and *erf74;erf75* lines lack the ROS burst in the early stages of heat and other stresses as a result of the lower expression level of the *RbohD* proteins [117,118]. Together, these studies suggest that *ERF74* acts as a switch to control the RBOHD-dependent mechanism to maintain H_2_O_2_ homeostasis and that the induction of a ROS-scavenging enzyme is dependent on the *ERF74*-*RBOHD*-ROS-signaling pathway [117,118]. Similarly, studies using the *erf6* mutants suggest that the AP2/ERF domain-containing TF *ERF6* regulates ROS signaling in Arabidopsis during heat and other abiotic stresses, indicating that ERFs regulate the stress tolerance by controlling ROS homeostasis in an RBOHD-dependent manner [118,119,120]. More recently, using ethylene signaling-defective mutants, the role of additional ethylene response factors, *ERF95* and *ERF97*, involved in the basal thermotolerance of Arabidopsis were uncovered, suggesting that *ERF95* and *ERF97* genetically function downstream of EIN3 and that the ectopic, constitutive expression of *ERF95* or *ERF97* increases the basal thermotolerance [121]. It has been reported that *ERF95* and *ERF97* regulate the heat-responsive genes and directly bind to the promoter of *HSFA2*, thus establishing a connection between ethylene and its downstream regulation in the basal thermotolerance of plants [120,121].

#### 3.2.4. Role of Salicylic Acid during High Temperatures

Salicylic acid has been reported to be a key signaling molecule in plants under high temperatures. Earlier studies suggest that SA is primarily involved in promoting the basal thermotolerance by inducing several *HSPs* [122]. The SA levels increase upon heat stress in plants, which were shown to improve the photosynthetic capacity by protecting the photosystem II (PSII) complex from higher levels of ROS during heat stress [123,124,125]. A few studies suggest that the exogenous application of SA increased the activity of antioxidant enzymes such as CAT and alleviated the heat-induced reduction in pollen viability and floret fertility by regulating the ROS level in developing anthers [126]. Another study showed that H_2_O_2_-mediated SA prevented the pollen abortion caused by heat stress [127]. In *Medicago sativa* L., SA treatments triggered the heat shock proteins in the organelles and improved the activity of PSII and altered the rate of electron transport, thereby reducing the damage caused by heat stress [128]. These findings indicate the role of SA in regulating the antioxidant defense system and improving the photosynthetic efficiency of plants, resulting in enhanced thermotolerance [126,127,128,129].

#### 3.2.5. Role of CKs during High Temperatures

CKs are primarily involved in plant growth and developmental functions, including regulating the organ size, a role in the meristem activity of shoots and roots, and branching [130]. Although the exact nature of CKs during high temperatures is yet to be clarified, a few studies suggest their role in adaptive mechanisms during heat stress. Earlier studies found that the CK contents are generally reduced during high temperatures [131]. Recent studies indicated that heat stress induces a rapid but transient increase in active CK contents, followed by its depletion, signifying that CKs could serve as primary receptors in temperature sensing and serve as the first signal for thermomorphogenesis [132,133]. Transcriptomic and proteomic studies in Arabidopsis have indicated a large overlap of CK- and heat-responsive transcriptomic changes [132,133,134]. The exogenous application of CKs induced heat shock responses by upregulating the heat shock proteins, regulating several phosphoproteins and increasing the activity of the antioxidant system [132,133,134]. In Arabidopsis, the application of INCYDE (2-fluoro-6-(3-methoxyphenyl) aminopurine), an inhibitor of CK oxidase/dehydrogenase (CKX) enzymes, inhibited cytokinin degradation and mildly promoted the heat stress tolerance [135]. Taken together, these studies highlight the positive role of CKs during the high temperature response in plants [134,135].

### 3.3. Hormone and ROS Crosstalk during High Temperatures Stress

The ROS and hormonal signaling are tightly synchronized to regulate plant growth and development (Figure 1) [22,23,24,25,26]. At their optimal levels, the ROS and hormones are involved in the various developmental process, including seed germination, apoptosis, stomatal responses, root hair growth and elongation, and lignin synthesis, and their coordination is required to induce stress responses during high or low temperatures [21,136].

Once induced by heat stress, hormones alter the ROS levels, primarily by activating RBOHs and/or by altering redox signaling [21,22,23,32]. Nevertheless, ROS have been shown to act both upstream and downstream of the hormones, and the interplay between them can act as an amplification loop to control the gene expression and induce heat and other abiotic stress responses [21,23,54,137,138]. In addition, ROS can mediate the signaling crosstalk between different hormones. For example, in response to heat stress in tomatoes, the ROS produced via RBOHs were shown to mediate an interaction between ABA and BRs, resulting in an improved heat stress tolerance [28].

The transcriptional regulation mediated by hormones and/or ROS play a key role in mediating the heat stress responses in plants. ABA is reported as one of the early response hormones during high temperatures. ABA regulates its key TFs, AREB/ABFs, to mediate the ABA-dependent gene expression, which, in turn, influences the ROS production or enhances the antioxidant capacity, depending on the nature and severity of stress (Figure 2) [93,139]. ROS can act as a hormonal response signal, with ABA-induced ROS bursts emerging as an example [34,140,141]. Thus, the ROS regulatory systems and hormone signaling events need to be strictly coordinated to fine-tune the heat stress responses [34,141]. During heat stress, ABA also mediates *HSP* accumulation that, in turn, acts as a molecular chaperone to mediate heat tolerance by enhancing the scavenging of ROS and inducing the expression of HS-responsive genes [70,134]. Grafting studies in cucumbers showed that ABA-induced *HSP* accumulation occurred in an apoplastic ROS-dependent manner, indicating that inhibiting ROS production impaired the *HSP* accumulation and heat tolerance in the presence of ABA [98].

Plant development and heat stress tolerance are highly dependent on the interaction between BRs and ROS signaling. For example, similar to ABA, the BRs regulator BZR1 directly binds to the promoter of *RBOH1*, and *RBOH1*-mediated ROS triggers programmed cell death (PCD) and tapetal cell degradation [142,143]. In line with these observations, tomato plants overexpressing BZR1 showed an enhanced production of apoplastic H_2_O_2_ that interacts and binds to the promoters of FERONIA (FER 2/3) receptor-like kinase, resulting in heat stress tolerance [144]. During high temperatures stress, ROS mediate the signaling crosstalk between hormones, regulating the gene expression and inducing stress tolerance [69,71]. For example, the study by Zhou et al. showed that BRs-induced ROS mediated an interaction between ABA and BRs by activating RBOHs and resulted in an improved stress tolerance to heat in tomatoes [28].

The exact mechanisms by which SA induces ROS or ROS generated in the cells triggers SA biosynthesis remains unidentified. However, earlier studies indicate that SA inhibits the catalase activity and disturbs the cellular redox homeostasis, thus resulting in a higher ROS accumulation [145]. On the other hand, higher levels of SA were observed in plants with sustained ROS production in peroxisomes or the chloroplast [146]. Taken together, these results suggest that peroxisomes or chloroplast ROS trigger SA biosynthesis, which is required for the defense response [146]. The mutant analysis of SA biosynthesis or catalase-deficient mutants suggest that ROS can also function upstream of SA biosynthesis, suggesting that ROS and SA function in a self-amplifying feedback loop [23,54]. For example, by analyzing the mutants deficient in SA signaling (*sid2*), it was shown that the mutants accumulated higher ROS levels and showed tolerance to a combination of heat and drought stress [129,147]. Another study suggested that the exogenous application of SA increased the heat tolerance in tomatoes and barley by improving the antioxidant defense system through the scavenging of ROS [148,149]. Like SA, CKs are also proven to enhance the heat tolerance in plants. Although previous studies have shown that heat inhibits the cytokinin levels by downregulating histidine kinases genes, treating plants with CK upregulated antioxidant system activity triggered *HSPs*, which lead to heat tolerance [133,134].

## 4. Low Temperatures Stress

Sudden or extremely low temperatures cause adverse effects on a plant’s metabolism, growth, and stress responses [150]. Some of the adverse effects of cold stress include membrane rigidification, the modification of stability and degradation of proteins, a higher accumulation of toxic metabolites, a reduced efficiency of ROS scavenging enzymes, and a lower photosynthesis rate [151]. Low temperatures negatively affect the gene expression, protein synthesis, and stress-responsive transcripts. At extreme low temperatures (<0 °C), ice crystals can form, which influences a plant’s water potential, leading to an osmotic imbalance and oxidative stress, thereby impacting the normal cellular and metabolic processes of the cell [152,153,154].

**Figure 3:**
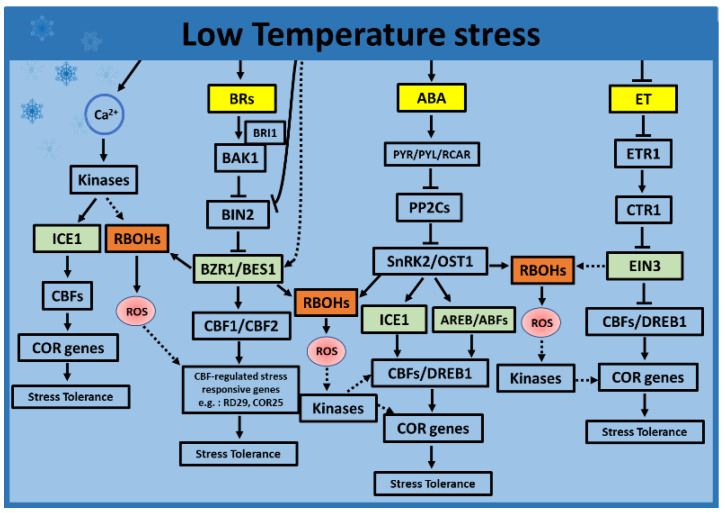
Selected hormone and ROS interactions in response to low-temperature stress. During low temperatures, ABA-regulated kinase SnRK2 regulates the stability and transcriptional activity of ICE1, causing the activation of CBF/COR. Parallelly, SnRK2 could phosphorylate RBOHs, generating ROS. Similarly, TFs BZR1 and EIN3 possibly induce RBOHs to generate ROS via the BR and ET-signaling pathways, respectively. The ROS generated in a feedback loop could induce the expression of CBFs via kinases. Alternatively, low temperatures cause membrane rigidification, which might induce a calcium signature that activates kinases. The activated kinases could phosphorylate ICE1 or RBOHs, thus inducing the expression of *COR* genes to orchestrate the appropriate molecular and physiological acclimation responses to low temperatures. Other plant hormones such as JA and SA may also play roles in inducing the stress responses to low temperatures. Only ABA, BRs, and ET were selected to be presented in the mode due to the availability of a relatively large amount of data supporting their roles in ROS interactions in response to low-temperature stress. Solid arrows and blocked arrows indicate confirmed positive and negative regulations. Dashed lines indicate potential interactions. The hormones are indicated in yellow, transcription factors in green, ROS in red, and the ROS-producing and -scavenging enzymes are indicated in brown and blue, respectively. ABA, abscisic acid; AREB/ABF, ABA-responsive element-binding protein/ABA-binding factor; BR, brassinosteroid; BAK1, BRI1-ASSOCIATED KINASE1; BES1, BRI1-EMS SUPPRESSOR1; BIN2, brassinosteroid insensitive 2; BRI1, BRASSINOSTEROID INSENSITIVE1; BZR1, BRASSINAZOLE-RESISTANT1; Ca^2+^, calcium; CBF, C-repeat-binding factor; *COR*, cold-response genes; CTR1, CONSTITUTIVE TRIPLE RESPONSE1; DREB1, dehydration response element binding factor1; ET, ethylene; EIN3, ETHYLENE INSENSITIVE3; ETR1, ethylene receptors; ICE1, inducer of CBF expression 1; OST1, OPEN STOMATA 1; PP2C, protein phosphatase 2Cs; PYR/PYL/RCAR, pyrabactin resistance protein or PYR-like proteins; RBOHs, respiratory burst oxidase homologs; ROS, reactive oxygen species; and SnRK2, SNF1-related protein kinase 2.

Plants have evolved sophisticated perception, signaling, and acclimation strategies that allow their survival at low temperatures, even at the cost of reduced growth and yield [155]. Changes in the lipid and protein compositions of the cellular membranes, the accumulation of cryoprotective polypeptides, soluble sugars, higher antioxidant enzymes, higher hormone accumulation, anti-freezing proteins (AFPs), and cold shock proteins (CSPs) are common at low temperatures [156,157,158]. Low temperatures are also sensed by changes in the membrane fluidity, such as the reduction of cell membrane fluidity and activate cold-responsive Ca^2+^ channels like mid1-complementing activity 1 and 2 (MCA1 and MCA2), or other Ca^2+^ signatures that result in a rise of the cytosolic Ca^2+^ levels [159]. The higher Ca^2+^ levels lead to the activation of RBOHs, thereby generating ROS. For example, a recent study in tomato plants suggested that CDPKs like CDPK27 induce crosstalk between ROS and other signaling molecules, leading to the activation of ABA. This suggests that CDPKs may function as a positive regulator of hormones in plant adaptation to cold stress [160]. It should also be noted that, apart from CDPKs, CaM-regulated receptor-like kinases 1 (CRLK1) via interactions with calmodulin (CaM) were shown to be important for plant responses to cold stress [160,161]. Thus, calcium, along with other secondary metabolites and signals, including ROS, NO, or hormones, mediate the electrophysiological responses and initiate cold stress responses [151,162,163]. In most plant species, the cold response is primarily mediated by the hormone-induced C REPEAT-BINDING FACTOR- COLD RESPONSIVE (*CBF-COR*) signaling pathway, where CBF directly binds to the promoters of the *COR* genes and induces their expression, thus enhancing the freezing tolerance (Figure 1) [118]. These CBFs, in turn, regulate the expression of the genes involved in ROS detoxification, hormone metabolism, and other presumed cellular protective functions [17]. Early molecular studies revealed that an MYC-type transcription factor INDUCER OF CBF EXPRESSION (ICE1) regulates the activity of *CBF/DREB1* genes [17,164], and these TFs and other downstream signaling components are regulated by a coordination between different hormones and ROS (Figure 1 and Figure 3).

### 4.1. Role of ROS during Low Temperatures Stress

Like in heat stress, low or freezing temperatures lead to ROS formation, and an excess accumulation of ROS in cell membranes induces oxidative stress. Low temperatures induce an enhanced rate of oxygenation reactions in the chloroplasts and produce a higher glycolate content. The glycolate is then converted to glyoxylate in the peroxisomes, producing H_2_O_2_ as a byproduct [89]. A higher accumulation of H_2_O_2_ results in activation of the ROS scavenging system through the conversion of GSH (reduced glutathione) to GSSG (oxidized glutathione) by the enzyme glutathione peroxidase (GPX) and glutathione S-transferase (GST), thus protecting plants from cold-induced oxidative stress [88,89].

In rice, the ROS-mediated gene expression and upregulation of antioxidant-related metabolites were shown to be involved in attaining chilling tolerance [165]. For example, metabolomic studies using rice varieties, such as *japonica*, *indica*, or nipponbare, at low temperatures showed a higher accumulation of MDA contents and H_2_O_2_ levels compared to their controls, revealing a ROS-dominated rice adaptation mechanism to low temperature environments [165]. Furthermore, the gene expression in response to cold stress may be regulated by redox signaling, as several defense genes containing antioxidant-responsive elements in their promoter regions [88]. More recently, the proteomics analysis in *Brassica napus* L. showed that the molecular mechanism of enhanced cold tolerance was achieved through ROS scavenging via the metabolic pathways [166].

### 4.2. Role of Hormones during Low Temperature Stress

#### 4.2.1. Role of ABA during Low Temperatures

As in other abiotic stress responses, cold stress responses are mediated by both the individual and combined actions of plant hormones. ABA is one of the key hormones involved in cold stress acclimation in plants [17]. ABA can induce an increase in the transcript levels of *CBF* genes by binding to the CRT/DRE element and activating *CBF* or induce *COR* gene expression [167]. All signaling components of the ABA signaling pathways were shown to be important players in attaining a cold tolerance. For example, OPEN STOMATA1 (OST1), an SNF1-related protein kinases2 (SnRK2) protein kinase family member, is a central regulator of the cold signaling pathway in Arabidopsis. The cold activated protein kinase SnRK2/OST1 phosphorylates ICE1, which prevents ICE1 degradation mediated by HIGH EXPRESSION OF OSMOTICALLY RESPONSIVE GENE15 (*HOS1*) and subsequently promotes the stability and binding of ICE to the CBF promoters, leading to *COR* gene expression, thereby enhancing the freezing tolerance (Figure 3) [168,169]. A mutant analysis indicated that the transgenic plants overexpressing OST1 exhibit an enhanced freezing tolerance, while the *ost1* mutants exhibited a freezing hypersensitivity [168]. In parallel to these observations, the crosstalk of ABA-induced OST1 along with MAPKs was also shown to phosphorylate ICE1 to induce cold stress responses [170]. Likewise, during cold or ABA treatments, a type 2C phosphatase ABA INSENSITIVE1 (ABI1) was shown to partially inhibit OST1, and the loss-of-function mutants of *abi1* showed an enhanced tolerance to freezing stress, highlighting the importance of protein phosphatases in inducing cold stress acclimation [168].

#### 4.2.2. Role of JA during Low Temperatures

During low temperatures, the synthesized JA activates the JA receptor COI1 (CORONATINE-INSENSITIVE 1) and promotes the degradation of JAZ (JASMONATE-ZIM-DOMAIN) proteins via the ubiquitin/26S proteasome pathway. The negative regulator of the JA-signaling pathway, JAZ1, physically interacts and represses the transcriptional function of ICE1, activating the *ICE-CBF* transcriptional regulation cascade, followed by the expressions of cold-regulated genes to improve the plant freezing tolerance [15,171,172]. In addition, the increased expression of JA biosynthesis and signaling genes were observed in rice plants when they were exposed to cold treatment [173]. In contrast, the plants showed a hypersensitive response to freezing stress when the JA signaling pathway was inhibited [174]. Experiments using apple seedlings indicated that MIEL1 (MYB30-Interacting E3 Ligase1) and JAZ proteins coregulated the JA-mediated cold stress tolerance via the B-box protein *BBX37-ICE1-CBF* module that aided in cold stress tolerance [174]. Furthermore, JA or its precursors such as OPDA or MeJA are well-known to induce ROS production, and plants treated with MeJA showed a higher antioxidant capacity, thus enhancing the cold tolerance. More recently, it was shown that cyclic nucleotide-gated channels and glutamate receptor-like channels (GLRs) play a role in inducing cold stress acclimation by increasing the endogenous jasmonate levels under cold stress [175,176]. The plant hormone GA promotes the accumulation of *DELLA*s that regulate the induction of *CBF3* through ICE1 via JAZs, suggesting a possible crosstalk between JA and GA in attaining an acclimation to cold [177].

#### 4.2.3. Role of ET during Low Temperatures

The plant hormone ET acts as a negative regulator of cold stress responses (Figure 3) [178]. In the ET signaling pathway, one of the primary negative regulators of the CBF expression is the TF ETHYLENE INSENSITIVE3 (EIN3). The EIN3 is degraded by two F-box proteins (EIN3-BINDING F-BOX 1 (EBF1) and EBF2) via the 26S proteasome pathway enhancing the stability of PHYTOCHROME-INTERACTING FACTOR 3 (PIF3), thus repressing the activity of *CBF* genes during cold stress [179,180]. A transcriptomic study using ERF mutants in Arabidopsis showed that two genes, *ERF102* and *ERF103,* are involved in the cold signaling pathway [181]. In addition, higher ROS accumulation in *erf105* plants suggests a role for *ERF105* in regulating ROS homeostasis, which is important for the cold stress response [17,182]. As in heat stress, miRNAs are also involved in cold stress tolerance via ET signaling. For example, reports on *Poncirus trifoliata* showed that ptr-miR396b positively regulates cold tolerance through reducing the *1-AMINOCYCLOPROPANE-1-CARBOXYLIC ACID OXIDASE* (ACO) transcript levels and repressing ethylene synthesis and leads to enhanced ROS scavenging, thus inducing cold tolerance [165].

#### 4.2.4. Role of BRs during Low Temperatures

In contrast to ET, the plant hormone BR positively regulates the freezing tolerance. Studies using BR signaling mutants in Arabidopsis showed that TFs BRZ1 and BES1 regulate the *CBF* expression by binding to the promoters of these genes, thus resulting in a freezing tolerance [108,183]. Since BRs are shown to be specific regulators of the *CBF-COR* pathway, these authors suggest that *CBF* genes may constitute a central node of hormone crosstalk during cold stress response [108]. In tomatoes, BRs were shown to positively regulate chilling tolerance via a signaling cascade involving *RBOH1*, GLUTAREDOXIN (*GRX*), and 2-Cys Prx. The BR-induced chilling tolerance was associated with an increase in the transcripts of the *RBOH1* and *GRX* genes, increasing the ratio of reduced/oxidized 2-cysteine peroxiredoxin (2-Cys Prx) and activation of the antioxidant enzymes [184]. Furthermore, in response to chilling stress, BRs were shown to act as positive regulators of photoprotection by inducing ROS production. The leucine-rich repeat receptor-like kinase, BZR1, induces the transcription of an NADPH oxidase-encoding gene, *RBOH1,* leading to a higher accumulation of apoplastic H_2_O_2_. The presence of ROS induces a cyclic electron flow, non-photochemical quenching (NPQ), accumulation of photoprotective proteins, and increased activity of several antioxidant enzymes, thus inducing photoprotection under low temperatures [30]. Furthermore, the BR-induced production of antioxidant enzymes aid in maintaining the ROS homeostasis and induces stress tolerance following cold stress [23,185]. The exogenous application of BRs leads to enhanced antioxidant capacity, orchestrating the alleviation of ROS burst-induced oxidative damage [186]. The exogenous application of BRs also increases the expression of *CBF* genes that control a significant portion of *COR* genes and are required for the adequate development of cold acclimation response [183,187].

### 4.3. Hormone and ROS Crosstalk during Low Temperatures Stress

Some of the primary hormones inducing stress responses to low temperatures include ABA and BRs (Figure 3). The signaling components of these hormones, OST1 or BZR1/BES1, regulate the TF activity of ICE1 and induce the expression of *CBF* genes. ABA and BRs are known to trigger H_2_O_2_ production via the RBOHs, resulting in enhanced cold tolerance and acclimation response [17,27,29,108]. A recent study demonstrated that the exogenous application of ABA enhanced cold tolerance by increasing the antioxidant enzymes like CAT, SOD, and APX [188]. Likewise, in a BR signaling pathway, BZR1 was shown to activate *RBOH1* resulting in the production of ROS [30,189]. In line with these observations, many studies indicated that priming plants with H_2_O_2_ improves plant’s antioxidant capacity and induces cold tolerance [27,190].

Cold stress increases the endogenous levels of JA in plants. During this process, the inducer of the *ICE1-CBF* regulatory cascade plays a key role in the regulation of cold stress tolerance [174]. JA has also been shown to counteract chilling stress by inducing ROS scavenging enzymes. Although the precise mechanism and pathways of the interaction between JA and ROS during low temperatures remains to be clarified, a few studies suggest that treatments of cold-stored lemon with MeJA together with SA increased its chilling tolerance by inhibiting the activity of polyphenol oxidase (PPO) and peroxidase (POD) [191,192,193].

Many ET-response TFs regulate ROS homeostasis and function in cold tolerance [194]. For instance, a study on ethylene-responsive factor in tobacco reported that overexpressing *MfERF1* showed higher activities of antioxidants such as Zn-SOD, CAT1, CAT2, and resulted in increased tolerance to freezing and chilling in transgenic tobacco, whereas KO mutants showed reduced freezing tolerance. ET also induces or transcriptionally inhibits the expression of *CBF/DREB1* via EIN3, a negative regulator of ethylene signaling (Figure 3) [178]. Parallel studies using *ERF109* from *Poncirus trifoliata* (L.) showed that *ERF109* binds to the promoter and regulates the expression of the POD-encoding gene (*PtrPrx1*), a class III peroxidase, to maintain a robust antioxidant capacity for effectively scavenging ROS, thus conferring cold tolerance [195]. Taken together, these studies highlight the role of hormones, ROS, and the intrinsic coordination between them, to induce tolerance to temperature stress. Plant hormones and ROS not only interact with each other, but also with other key signaling components, including NO, Ca^2+^, kinases, phosphatases, TFs, and various other metabolites, to orchestrate appropriate molecular, metabolic, and physiological acclimation responses that regulate acclimation responses to high or low-temperature stress.

## 5. Conclusions and Perspectives

To overcome the effects of temperature fluctuations, plants have evolved sophisticated acclimation mechanisms, including signal transduction pathways that activate stress-specific TFs, genes, signaling molecules, and enhance antioxidant activity. Plant hormones are developmental regulators that control many cellular, biochemical, and physiological responses throughout the plant life cycle. Stress perception by receptors triggers changes in the hormone levels, which activate stress-specific signal transduction cascades to regulate the expression of stress-specific TFs and their related genes. Part of their response also includes interaction with other hormones and many signaling molecules, including ROS. Hormones trigger ROS production (via RBOHs), and ROS, in turn, can impact hormone biosynthesis and allocation. The variations in the pattern of ROS/hormone signatures after early stages of stress are known to be one of the key processes influencing different stress response networks, thus attaining stress acclimation. Hence, identifying these hormone-ROS-regulated signaling pathways will aid in a better understanding of the molecular mechanism and help in developing improved stress-tolerant plants.

During temperature fluctuations, one of the most toxic byproducts of the plant’s metabolism is the generation of ROS. Paradoxically, ROS also serve as signaling molecules involved in the long-distance signaling and stress acclimation process. Due to its metabolic and toxic nature, many studies suggest that genetic manipulation of genes involved in ROS metabolism or antioxidants and scavenging enzymes could increase tolerance to temperatures stress. Alternatively, insights into the mechanism of thermal tolerance through high-throughput techniques point towards the importance of exogenous application of lower concentrations of ROS or stress-specific hormones to induce higher sustainability during extreme temperatures. Engineering plants to synthesize these compounds may offer an alternate way of increasing thermotolerance among important crop species. Thus, identifying the role of ROS and hormone integration in the avenue of stress combination is an area of potential future research. Increasing evidence shows that priming plants with heat can improve their photosynthetic capacity, stomatal conductance, and develop a higher antioxidant capacity to avoid ROS-induced damages. Lastly, biotechnology approaches including genome-wide association studies, quantitative trait loci mapping, transcriptomics, metabolomics, and proteomics, followed by selective breeding or genetic engineering, can greatly aid in identifying the nature of the signaling cascades and the utilization of specific genes in coping with high or low temperatures. Once these molecular principles have been established, conventional plant breeding approaches including backcrossing and pedigree, or population breeding can be employed to develop high-yielding and thermotolerant plants that can adapt to climate change.

With the rapid changes in global temperatures, temperature stress is expected to be one of the primary abiotic stresses determining plant health. Temperatures, either high or low, and in their extremes, adversely affect all developmental processes in plants, from seed germination to cell death. They also negatively influence the plant’s physiological processes, including transpiration, photosynthesis, respiration, and cellular structures. At the cellular level, they disrupt various signal transduction pathways and generate and alter the levels of toxic metabolites, resulting in lower crop yield or death. Plant responses to these temperature fluctuations include stress perception via thermal sensors, alterations in the signaling pathways affecting plant redox and hormone levels, and changes in the activity of stress-specific TFs and genes. Many studies have independently identified various mechanisms that govern a plant’s response to temperature changes. However, a complete understanding of temperature stress tolerance mechanisms involving plant hormones and secondary metabolites such as ROS had remained elusive. Here we have provided a comprehensive view of recent work on the roles of plant hormones, ROS, and their integration in achieving plant tolerance to high and low temperatures.

## Notice

This manuscript has been authored by UT-Battelle, LLC under Contract No. DE-AC05-00OR22725 with the U.S. Department of Energy. The United States Government retains, and the publisher, by accepting the article for publication, acknowledges, that the United States Government retains a nonexclusive, paid-up, irrevocable, worldwide license to publish or reproduce the published form of this manuscript, or allow others to do so, for United States Government purposes. The Department of Energy will provide public access to these results of federally sponsored research in accordance with the DOE Public Access Plan (http://energy.gov/downloads/doe-public-access-plan accessed on 18 July 2021).
